# Royal Jelly Exerts a Potent Anti-Obesity Effect in Rats by Activating Lipolysis and Suppressing Adipogenesis

**DOI:** 10.3390/nu16183174

**Published:** 2024-09-19

**Authors:** Alaa Hasanain Felemban, Ghedeir M. Alshammari, Abu ElGasim Ahmed Yagoub, Ali Saleh, Mohammed Abdo Yahya

**Affiliations:** Department of Food Science and Nutrition, College of Food and Agricultural Science, King Saud University, Riyadh 11451, Saudi Arabia; alaa.felemban@gmail.com (A.H.F.); amohammed4@ksu.edu.sa (A.E.A.Y.); 442106909@student.ksu.edu.sa (A.S.); mabdo@ksu.edu.sa (M.A.Y.)

**Keywords:** royal jelly, obesity, adipogenesis, lipolysis, AMPK, SREBP1, PPARγ

## Abstract

**Background/Objective:** This study examined the anti-obesity effect of royal jelly (RJ) in rats fed with a high-fat diet by targeting the major pathways involved in adipogenesis and lipolysis. In addition, it examined whether this effect is AMPK-dependent. **Methods:** Five groups of adult male albino rats were used (*n* = 6 each as 1); the control rats were fed with a normal diet (2.9 kcal), and the other groups were as follows: control + RJ (300 mg/kg), HFD (4.75 kcal), HFD + RJ (300 mg/kg), and HFD + RJ (300 mg/kg) + dorsomorphin (an AMPK inhibitor) (0.2 mg/kg). **Results:** RJ was administered orally to all rats. With no changes in food and energy intake, RJ significantly reduced gains in body weight, fat weight, body mass index (BMI), the Lee index, abdominal circumference (AC), and the adiposity index (AI). It also reduced fasting glucose and insulin levels, HOMA-IR, and the circulatory levels of free fatty acids (FFAs), triglycerides, cholesterol, and LDL-c in the HFD-fed rats. RJ also increased serum glycerol levels and adiponectin levels, but reduced the serum levels of leptin, IL-6, and TNF-α. Moreover, RJ reduced the secretion of IL-6 and TNF-α from isolated WAT. At the tissue level, the HFD + RJ rats exhibited a smaller adipocyte size compared to the HFD rats. At the molecular level, RJ increased the phosphorylation of AMPK, SREBP1, and ACC-1 and increased the mRNA and protein levels of HSL and ATG in the WAT of the HFD rats. In concomitance, RJ increased the mRNA levels of PGC-α1, reduced the protein levels of PPARγ, and repressed the transcriptional activities of PPARγ, SREBP1, and C/EBPαβ in the WAT of these rats. All the aforementioned effects of RJ were prevented by co-treatment with dorsomorphin. **Conclusions:** RJ exerts a potent anti-obesity effect in rats that is mediated by the AMPk-dependent suppression of WAT adipogenesis and the stimulation of lipolysis.

## 1. Introduction

Obesity represents a chronic metabolic disorder characterized by the excessive accumulation of adipose tissue, resulting from an imbalance between caloric intake and energy expenditure [1]. This condition is marked by the proliferation and dysfunction of white adipose tissue (WAT), alongside aberrant fat deposition in critical organs such as the liver, skeletal muscle, kidneys, and heart [1,2]. The pathological consequences of obesity extend to an increased susceptibility to serious health conditions, including dyslipidemia, insulin resistance (IR), type 2 diabetes mellitus (T2DM), hypertension, stroke, myocardial infarction (MI), and various cancers [1,2]. Globally, obesity has escalated to pandemic proportions, imposing significant burdens on healthcare systems and economic structures. Recent data indicate that approximately 38% of the global population is either overweight or obese, with projections suggesting that this figure could surge to 51% by 2035 [1,2]. In the United States, estimates predict that 78% of adults will experience obesity by 2030 [2,3]. In Saudi Arabia, a 2019 WHO survey reported that 38% of the population is overweight and 20% is obese. Given these alarming statistics, it is imperative to prioritize the development of effective strategies for the prevention and management of obesity.

Understanding the mechanisms regulating the lipid metabolism within adipose tissue is essential for advancing therapeutic and preventive measures. Adipose tissue homeostasis is fundamentally maintained through a balance between lipid accumulation and mobilization. Adipogenesis, the process by which pre-adipocytes in WAT differentiate into mature adipocytes capable of lipid storage, involves intricate signaling pathways and the activation of transcription factors such as cyclic AMP response element-binding protein (CREB), CCAAT/enhancer-binding proteins (C/EBPα and C/EBPβ), adipocyte fatty acid-binding protein (aP2), peroxisome proliferator-activated receptor-γ (PPARγ), and sterol regulatory element-binding protein-1c (SREBP-1c) [4,5]. Conversely, lipolysis, the hydrolysis of triglycerides into fatty acids and glycerol, is mediated by key enzymes including hormone-sensitive lipase (HSL) and adipose triglyceride lipase (ATGL) [6,7]. A disruption in the equilibrium between adipogenesis and lipolysis leads to adipose tissue hypertrophy, hyperplasia, dysfunction, and inflammation, exacerbating obesity-related IR and T2DM [8,9,10]. Promoting lipolysis or inhibiting adipogenic pathways can mitigate obesity and its associated metabolic disorders [11,12,13,14,15].

Furthermore, obesity and adipogenesis are linked to alterations in various signaling pathways within the WAT, with 5’-adenosine monophosphate (AMP)-activated protein kinase (AMPK) emerging as a pivotal regulator due to its anti-adipogenic effects across multiple tissues, including the liver and skeletal muscle [16,17]. AMPK, a serine/threonine kinase sensitive to cellular ATP levels, functions as a key regulator of the lipid and glucose metabolisms, with recent research highlighting its role in modulating adipogenesis, lipolysis, thermogenesis, and fatty acid oxidation. Notably, AMPKα2 knockout mice exhibit obesity, enhanced adipose tissue expansion, diminished thermogenesis, and compromised glucose tolerance [3,18,19,20]. In overweight and obese individuals, AMPK expression and activity are markedly reduced [3,21,22,23]. The activation of AMPK through agents such as metformin, R-17, BC-1618, MK-3903, DNP-60502, A-769662, and Ampkinone has been shown to alleviate obesity, improve glucose tolerance, and mitigate IR by suppressing adipogenesis, reducing inflammation, and promoting lipolysis, mitochondrial biogenesis, thermogenesis, autophagy, and fatty acid oxidation [3,24,25,26,27,28,29,30]. Moreover, AMPK activation inhibits adipocyte differentiation and stimulates lipolysis in vitro [31,32,33,34]. The therapeutic potential of targeting AMPK for obesity management is, thus, well-supported by its regulatory influence on enzymes and transcription factors, including acetyl CoA carboxylase (ACC), fatty acid synthase (FAS), PGC-1α, SREBP-1, PPARγ, C/EBPα, and C/EBPβ [3,24,35].

Traditional medicine and natural products, such as royal jelly (RJ), offer additional avenues for obesity management by modulating AMPK activity [36,37,38,39,40]. RJ, a nutrient-rich secretion produced by worker honeybees for larval and queen nourishment, is recognized for its diverse pharmacological properties, including anti-aging, antioxidant, anti-inflammatory, antimicrobial, antidiabetic, anti-hypercholesterolemic, anticancer, and wound-healing effects [41,42,43,44]. RJ has demonstrated efficacy in regulating body composition, mitigating muscle lipotoxicity, enhancing insulin sensitivity, and reducing adipose tissue mass and body weight in both human and animal models of obesity [45,46,47,48,49]. However, the detailed molecular mechanisms through which RJ exerts its anti-obesity effects remain inadequately characterized. Initial studies have suggested that RJ promotes thermogenesis and modulates adipocyte differentiation by influencing transcription factors such as C/EBPα, PPARγ, and SREBP-1c [48]. Additionally, RJ has been shown to activate AMPK in animal models, reducing hepatic gluconeogenesis and ameliorating non-alcoholic fatty liver disease by enhancing insulin sensitivity, suppressing hepatic lipogenesis, and stimulating fatty acid oxidation via AMPK-dependent pathways [50,51]. The involvement of AMPK in these processes underscores its potential as a therapeutic target for obesity.

In light of these findings and the current lack of comprehensive in vivo studies, our research investigates the hypothesis that RJ administration can alleviate obesity in high-fat diet (HFD)-fed rats by inhibiting adipogenesis and promoting lipolysis and fatty acid oxidation, primarily through AMPK activation. The impact of RJ on AMPK expression and its related transcription factors will be assessed in the presence or absence of an AMPK inhibitor to elucidate the underlying mechanisms of its anti-obesity effects.

## 2. Materials and Methods

### 2.1. Animal Subjects

For this investigation, a total of 30 male Wistar rats, each at six weeks of age and weighing approximately 120 ± 10 g, were sourced from the Experimental Animal Care Center at King Saud University, Riyadh, Saudi Arabia, with prior approval from the Research Ethics Committee (REC) (Ethical Reference No: KSU-SE-22-52). Initially, these rats were accommodated in groups of six within standard housing units, allowing for acclimatization to the controlled environment. The housing conditions were meticulously regulated, maintaining a constant temperature of 22.5 ± 1 °C and a relative humidity of 55–60%. The light cycle was standardized with illumination from 6:00 a.m. to 6:00 p.m. During the adaptation period, the rats had unrestricted access to water and a standard diet (13% fat; Cat. #D10012M, Research Diets, New Brunswick, NJ, USA). The cages were furnished with wood shavings and paper wool to enhance environmental enrichment. All animal care procedures and experimental interventions were overseen by qualified veterinarians from the facility.

### 2.2. Dietary Interventions

The standard control diet (Cat. #D10012M, Research Diets, NJ, USA) used in this study provided an energy density of 2.9 kcal/g (12.1 kJ/g), composed of 13% fat, 20% protein, and 67% carbohydrates. To induce obesity, the high-fat diet (HFD) (Cat. #D12451, Research Diets, New Brunswick, NJ, USA) was employed, which offered an energy density of 4.73 kcal/g (19.8 kJ/g) with 45% fat, 20% protein, and 35% carbohydrates. This HFD is specifically designed to induce obesity and Type 2 Diabetes Mellitus (T2DM) in rodent models and has been well-documented in previous research [52,53,54,55]. This diet has also been utilized to induce non-alcoholic fatty liver disease (NAFLD) in rats and assess the impact of royal jelly (RJ) on NAFLD in obese rat models [51].

### 2.3. Royal Jelly Preparation

The royal jelly (RJ) used in this study was procured from Batterjee Pharmaceutical Factory, Jeddah, Saudi Arabia. The RJ was freshly harvested from local beekeepers during the summer of 2023 and purchased in its natural state, free from any additives or preservatives. It was processed and stored under conditions optimized to preserve its natural characteristics and ensure its purity. Quality control measures verified the absence of any additives, ensuring that the RJ maintained its typical composition and seasonal quality. Each gram of RJ provided an energy content of 2.9 kcal.

### 2.4. Experimental Protocol

The experimental framework was modeled based on our previous study, where the daily administration of RJ at a dosage of 300 mg/kg over a 12-week period significantly reduced body weight, ameliorated insulin resistance and hyperglycemia, and decreased the mass of subcutaneous and mesenteric fat in rats fed with an HFD [51]. Following a one-week acclimatization phase, the rats were allocated into five distinct groups (*n* = 6 per group): (1) a control group receiving the standard control diet and normal saline (vehicle); (2) an HFD group receiving the D12451 HFD and normal saline; (3) a control + RJ group consuming the control diet and treated with RJ (300 mg/kg); (4) an HFD + RJ group on the D12451 HFD and treated with RJ (300 mg/kg); and (5) an HFD + RJ + dorsomorphin group, similar to Group 4, but with the additional receipt of dorsomorphin (0.2 mg/kg i.p.) administered 2 h before RJ. Dorsomorphin, also known as compound C, is a selective AMPK inhibitor, and this dosage has been validated in prior studies for effective in vivo AMPK inhibition across various tissues [51,56,57,58,59]. Preliminary data also confirmed its efficacy in suppressing the AMPK activity in adipose tissue.

### 2.5. Nutritional and Anthropometric Measurements

Nutritional intake and anthropometric parameters were systematically assessed every four weeks throughout the study, employing established methodologies [60,61,62]. The following equations were used for the calculations: (1) Total energy intake (kJ/day) = (the average food consumption × the dietary metabolizable energy). (2) Body mass index (BMI) = [body weight (g) ÷ length^2^ (cm^2^)]. (3) Lee index (%) = $\left(\sqrt[{3}]{body}weight\left(g\right)÷length\left(cm\right)\times 1000\right)$. The length was measured from the nose tip to the beginning of the tail. Values of the Lee index (%) > 310 confirm obesity in rats [62]. (4) The abdominal circumference (AC) = area circumference immediately anterior to the forefoot. All measurements were conducted for *n* = 6 rats/group.

### 2.6. Blood and Adipose Tissue Collection and Adiposity Index Calculation

After a 14 h fasting period, the rats were anesthetized with ketamine (80 mg/rat). Blood samples were obtained via cardiac puncture and collected into either plain or EDTA tubes. Concurrently, fat pads (subcutaneous, parametrial, mesenteric, perirenal, and retroperitoneal) were excised on ice. These were initially weighed, rinsed with phosphate-buffered saline (pH = 7), and subsequently stored at −80 °C. The adiposity index (AI) was calculated using the formula: [AI (%) = (total fat pad weight ÷ final body weight (g)) × 100]. The AI was assessed for *n* = 6 rats per group.

### 2.7. Serum Lipid and Adipokine Analysis

At the conclusion of the 12-week period, the rats were fasted for 12 h and then anesthetized with a ketamine/xylazine mixture (80/10 mg/kg). Blood samples (1 mL) were collected via cardiac puncture and processed into plasma and serum. Glucose and insulin levels were measured using specific assay kits (Cat No. 10009582, Cayman Chemicals, Ann Arbor, CA, USA; Cat. No. 589501, Ann Arbor, CA, USA). Insulin resistance was evaluated using the homeostasis model assessment (HOMA), calculated by the formula: glucose (mg/dL) × insulin (ng/mL)/405. The serum levels of triglycerides (TG), free fatty acids (FFAs), cholesterol, low-density lipoprotein (LDL), and high-density lipoprotein (HDL) were measured using commercial colorimetric assays (Cat. #ECCH-100, BioAssay Systems, Hayward, CA, USA; Cat. #MBS014345, MyBioSource, San Diego, CA, USA; Cat. #10009582, Cayman Chemicals, Ann Arbor, MI, USA; Cat. #79960, Crystal Chemicals, Houston, TX, USA; Cat. #STA-394, Cell Biolabs, San Diego, CA, USA). All assays were conducted in duplicate for *n* = 6 samples per group.

### 2.8. Adipokine and Free Glycerol Quantification

The serum concentrations of adiponectin and leptin were quantified using rat-specific ELISA kits (Cat. #RRP300, Cat. #MOB00B, R&D System, Minneapolis, MN, USA). Free glycerol levels were assessed with a colorimetric assay kit (ab65337, Abcam, Cambridge, UK). Each assay was performed in duplicate, with *n* = 6 samples per group.

### 2.9. Cytokine Release from Adipose Tissue

Cytokine release assays for TNF-α and IL-6 were performed as per Kern et al. [63]. Fresh adipose tissue samples were minced and incubated in serum-free Dulbecco’s Modified Eagle Medium (DMEM) with 10 mM HEPES buffer. After 24 h, the medium was analyzed for TNF-α and IL-6 levels using ELISA kits (Cat. #R6000B and Cat. #RTA00-1, R&D System, MN, USA). Analysis was conducted in triplicate for *n* = 6 samples/group.

### 2.10. Protein Extraction from Adipose Tissue

The total proteins were extracted from 500 mg of frozen adipose tissue using the minute total protein isolation kit (Cat. #: AT-022, Invent Biotechnologies, Plymouth, MN, USA), which provides a high purity with minimal lipid contamination. Nuclear proteins were isolated using the minute nuclei isolation kit (Cat. #AN-029-IB, Invent Biotechnologies, MN, USA). The isolated nuclear fraction was then processed in 155.5 μL of extraction buffer (20 mM HEPES, 25% glycerol, 0.42 M NaCl, 0.2 mM EDTA, 1.5 mM MgCl_2_, pH 7.9) with the addition of 1.5 µL of 0.1 M DTT solution. To avoid protein degradation and phosphatase activation, 1.5 µL of a protease inhibitor cocktail (Cat. #ab271306, Abcam, Cambridge, UK) and 1.5 µL of a phosphatase inhibitor cocktail (Cat. #78420, ThermoFisher, Waltham, MA, USA) were incorporated during extraction. The protein concentrations in the cytoplasmic and nuclear fractions were measured using the Pierce assay kit (Cat. #23225, ThermoFisher). All fractions were stored at −80 °C until further analysis.

### 2.11. Biochemical Analysis of Adipose Tissue Proteins

Rat-specific ELISA kits were utilized to measure the levels of interleukin 6 (IL-6), tumor necrosis factor-alpha (TNF-α), fatty acid synthase (FAS), SREBP1, phosphorylated SREBP1 (Ser439), PPARγ, PGC-1α, AMPK, phosphorylated AMPK, acetyl-CoA carboxylase (ACC), phosphorylated ACC, hormone-sensitive lipase (HSL), and adipose triglyceride lipase (ATGL) in adipose tissue protein extracts (Cat. #R6000B, R&D System, MN, USA; Cat. #RTA00-1, R&D System, MN, USA; Cat. #MBS706221, MyBiosource, San Diego, CA, USA; Cat. #MBS1600312, MyBiosource, CA, USA). These assays were performed following standard protocols and were analyzed in duplicate for each sample.

### 2.12. Biochemical Analysis of Nuclear Adipose Tissue Fractions

To assess the transcriptional activities of key nuclear factors—C/EBPα/β, SREBP1, and PPARγ—in adipose tissues, we utilized rat-specific colorimetric and ELISA-based assay kits (Cat. #ab 207199, Cat. #ab133125, and Cat. #ab133101, respectively). Each assay was executed in duplicate for samples derived from six rats per experimental group.

### 2.13. Real-Time PCR Analysis

RNA was extracted from epididymal fat using the RNeasy Lipid Tissue Mini Kit (Cat.#74804, Qiagen, Hilden, Germany). Complementary DNA (cDNA) synthesis was performed with a commercial kit (Cat. No. K1621, Thermo Fisher). Gene-specific primers for AMPK, PPARγ, SREBP1, ATGL, HSF, and FAS were selected from the existing literature [40] and validated through PubMed primer design tools. Quantitative PCR (qPCR) was conducted with the SsoFast EvaGreen Supermix (Cat. No. 172-5200, Bio-Rad, Des Plaines, IL, USA) on a CFX96 real-time PCR system (Bio-Rad). The gene expression levels were normalized to β-actin, and qPCR assays were carried out with six samples per group.

### 2.14. Statistical Evaluation

Data analysis was performed using the GraphPad Prism software (version 8, GraphPad Software, LLC, SanDiego, CA, USA). Statistical comparisons of the data were conducted using a two-way ANOVA followed by Tukey’s post hoc test. Statistical significance was established at *p* < 0.05, and the results are reported as means ± standard deviation (SD).

## 3. Results

This section is divided by subheadings. It provides a concise and precise description of the experimental results, their interpretation, as well as the experimental conclusions that can be drawn.

### 3.1. RJ Attenuates the Gain in Body Weight and Decreases Adiposity Markers in HFD-Fed Obese Rats without Altering Food and Energy Intake

**Figure 1:**
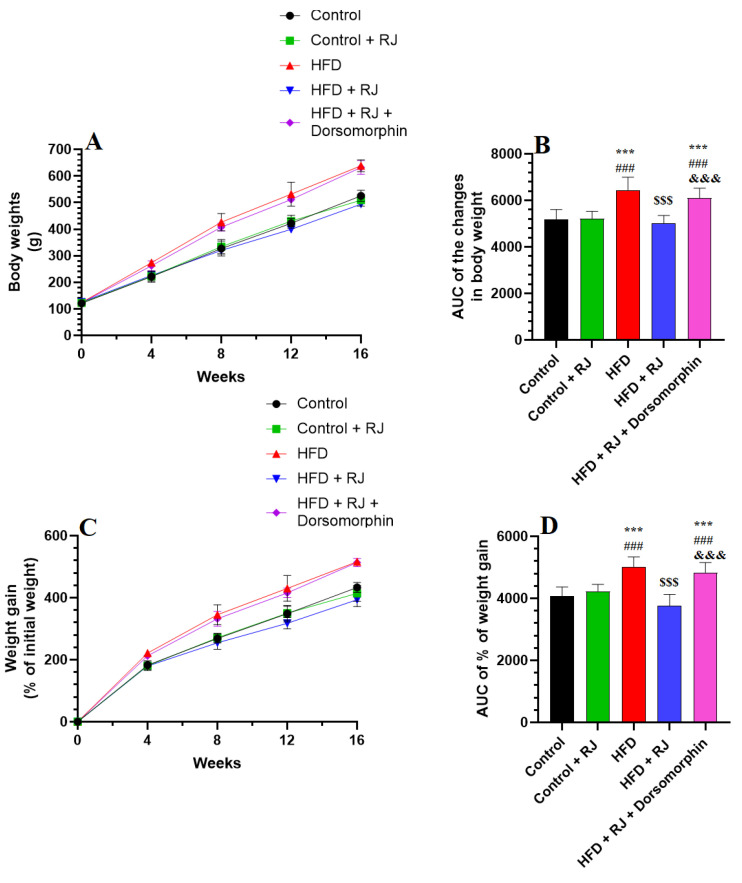
Alterations in body weights (**A**), AUC of body weight (**B**), weight gain (**C**), and AUC of weight gain (**D**) among all groups of rats. Data are given as means ± SD of 8 rats/group. ***: significantly different vs. control (*p* < 0.001); ###: significantly different vs. control + RJ (*p* < 0.001); $$$: significantly different vs. HFD (*p* < 0.001); and &&&: significantly different vs. HFD+ RJ (*p* < 0.001). RJ: royal jelly; HFD: high-fat diet. Dorsomorphin: an AMPK inhibitor.

**Figure 2:**
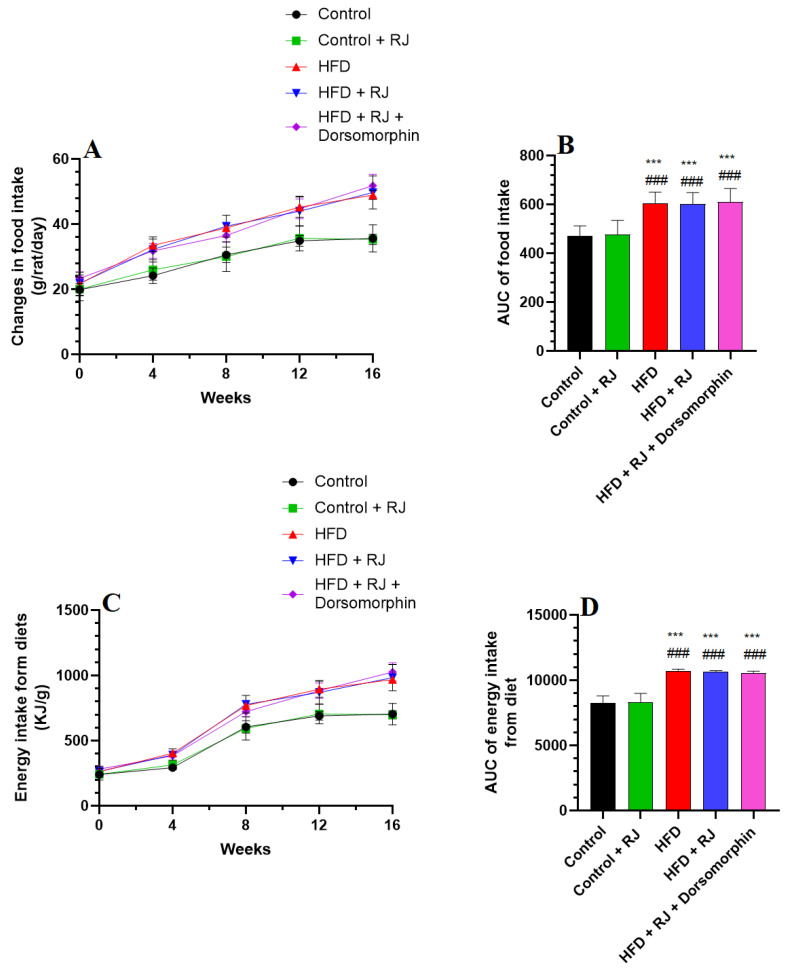
Alterations in food intake (**A**), AUC of food intake (**B**), energy intake (**C**), and AUC of energy intake (**D**) among all groups of rats. Data are given as means ± SD of 8 rats/group. ***: significantly different vs. control (*p* < 0.001) and ###: significantly different vs. control + RJ (*p* < 0.001). RJ: royal jelly; HFD: high-fat diet. Dorsomorphin: an AMPK inhibitor.

Energy intake was assessed exclusively from the diets, excluding the caloric contributions from the RJ. Changes in body weight, food and energy intake, and adiposity markers were evaluated across all experimental groups of rats, as detailed in Figure 1 and Figure 2 and Table 1 and Table 2. Rats fed with an HFD exhibited marked increases in their weekly body weight, food consumption, energy intake, final body weight, BMI, Lee index, and AC compared to the control group (Figure 1 and Figure 2; Table 1). Additionally, the HFD-fed rats displayed elevated weights of their mesenteric, retroperitoneal, perirenal, and subcutaneous fat pads, along with a higher AI relative to the control rats. In contrast, no significant differences in these parameters were observed between the control rats and those treated with the RJ.

However, rats subjected to the HFD with RJ treatment showed notable reductions in their weekly weight gain, final body weight, fat pad weights, BMI, AC, and AI compared to those on the HFD alone (Figure 1 and Figure 2; Table 1 and Table 2). Despite this, the food and caloric intake remained similar between the HFD and HFD + RJ groups. Furthermore, the administration of dorsomorphin, a selective AMPK inhibitor, effectively negated the beneficial effects of RJ in the HFD + RJ group, restoring the body weight, weight gain, food and energy intake, fat pad weights, BMI, AC, and AI to levels comparable with those observed in the HFD group (Figure 1 and Figure 2; Table 1 and Table 2).

### 3.2. RJ Attenuates the Impairment in Glucose/Lipid Metabolism and Adipokines’ Release and Increases Markers of Lipolysis in the Serum of HFD Obese Rats

As outlined in Table 3, compared to the control rats, the HFD-obese rats exhibited elevated plasma levels of fasting glucose and insulin, as well as increased HOMA-IR. These rats also demonstrated significantly higher levels of TGs, CHOL, LDL-c, leptin, glycerol, and FFAs (Table 3). Conversely, the adiponectin levels were significantly lower in the HFD-obese rats compared to the controls.

The control rats treated with RJ showed marked reductions in TGs, CHOL, FFAs, and LDL-c, alongside significantly higher levels of glycerol and adiponectin relative to the control rats without RJ treatment (Table 3). In the HFD + RJ-treated rats, the plasma levels of fasting glucose and insulin, along with HOMA-IR, TGs, CHOL, FFAs, and leptin, were significantly lower, while the serum levels of glycerol and adiponectin were significantly elevated compared to the controls (Table 3).

However, the inclusion of dosomorphin in the HFD + RJ regimen did not significantly alter the levels of these markers or chemical endpoints compared to the HFD + RJ group, nor did it differ significantly from the HFD model group (Table 3).

### 3.3. RJ Suppresses Levels of Inflammatory Cytokine Synthesis and Release from Adipose Tissue

**Figure 3:**
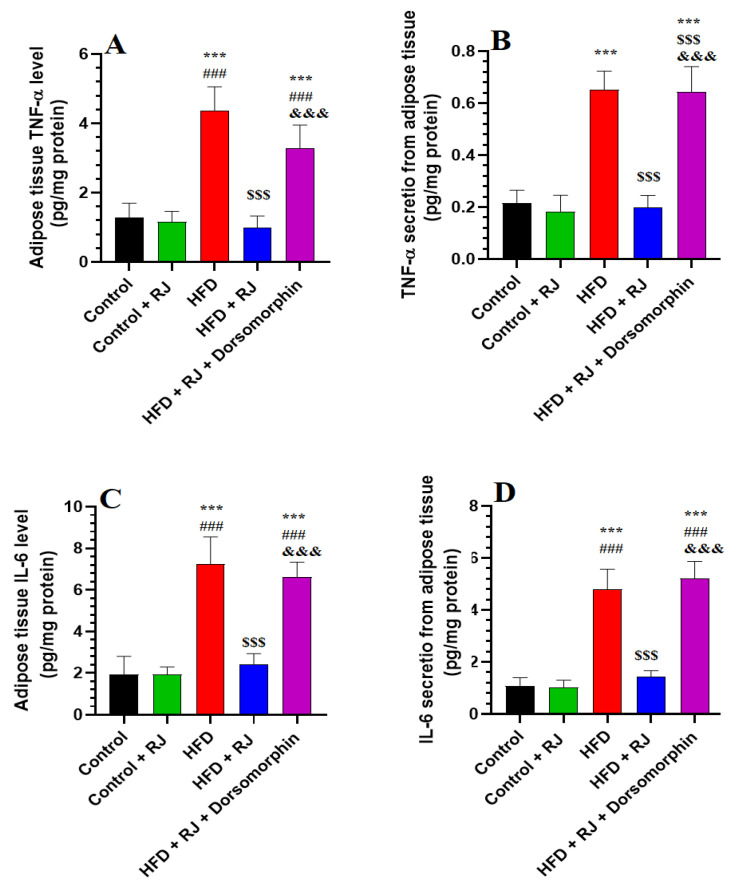
Levels of TNF-α (**A**,**B**) and IL-6 (**C**,**D**) in the white adipose tissue (WAT) collected from rats or secreted after being cultured in vitro. Data are given as the means ± SD of 6 rats/group. ***: significantly different vs. control (*p* < 0.001); ###: significantly different vs. control + RJ (*p* < 0.001); $$$: significantly different vs. HFD (*p* < 0.001); and &&&: significantly different vs. HFD + RJ (*p* < 0.001). RJ: royal jelly; HFD: high-fat diet. Dorsomorphin: an AMPK inhibitor.

No significant differences were observed in the adipose tissue levels of IL-6 and TNF-α between the control rats and those treated with RJ (Figure 3A,C). Similarly, the in vitro media levels of IL-6 and TNF-α released from the WAT of both the control and RJ-treated rats showed no statistical variation (Figure 3B,D). In contrast, the WAT and media levels of these cytokines were significantly elevated in the HFD-obese rats compared to the controls, but were significantly reduced in the HFD + RJ-treated group (Figure 3A–D). The levels of TNF-α and IL-6 in the WAT and culture media of the HFD + RJ + dorsomorphin-treated rats were significantly higher than those in the HFD + RJ group, yet did not differ significantly from the levels observed in the HFD group (Figure 3A–D).

### 3.4. RJ Activates AMPK and Suppresses ACC in the Adipose Tissue of HFD-Fed Obese Rats

**Figure 4:**
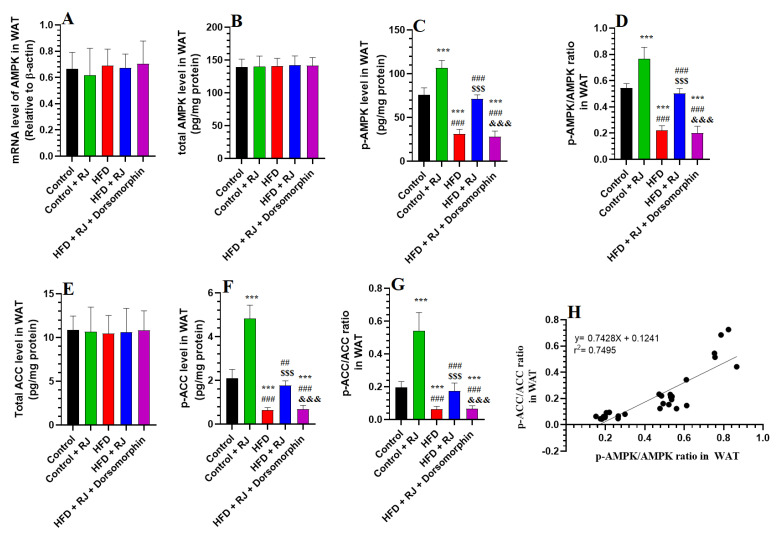
mRNA levels of AMPK, total protein levels of AMPK, phosphorylation levels of AMPK (**A**–**D**) and acetyl CoA carboxylase (ACC), the phosphorylation levels of ACC (**E**–**H**) in the white adipose tissue (WAT) of all groups of rats. Data are given as the means ± SD of 6 rats/group. ***: significantly different vs. control (*p* < 0.001); ^##^, ^###^: significantly different vs. control + RJ (*p* < 0.001); ^$$$^: significantly different vs. HFD (*p* < 0.001); and ^&&&^: significantly different vs. HFD + RJ (*p* < 0.001). RJ: royal jelly; HFD: high-fat diet. Dorsomorphin: an AMPK inhibitor.

No significant differences were found in the WAT mRNA and total levels of AMPK and ACC across all rat groups (Figure 4A,E). However, the phosphorylation levels of AMPK and ACC, along with the ratios of p-AMPK/AMPK and p-ACC/ACC, were significantly lower in the WAT of the HFD-fed obese rats compared to the control rats (Figure 4B–G). These phosphorylation levels were notably elevated in the WAT of the HFD + RJ-treated rats relative to the HFD group (Figure 4B–G). Despite this, the levels of p-AMPK, p-ACC, p-AMPK/AMPK, and p-ACC/ACC in the WAT of the HFD + RJ + dorsomorphin-treated rats were significantly lower than those in the HFD + RJ group and did not differ significantly from the levels in the HFD group (Figure 4B–G). Notably, a correlation analysis demonstrated a strong association between the WAT levels of p-AMPK and p-ACC (r^2^ = 0.7495) (Figure 4H).

### 3.5. RJ Attenuates the Increment in Transcription Factors of Adipogenesis

**Figure 5:**
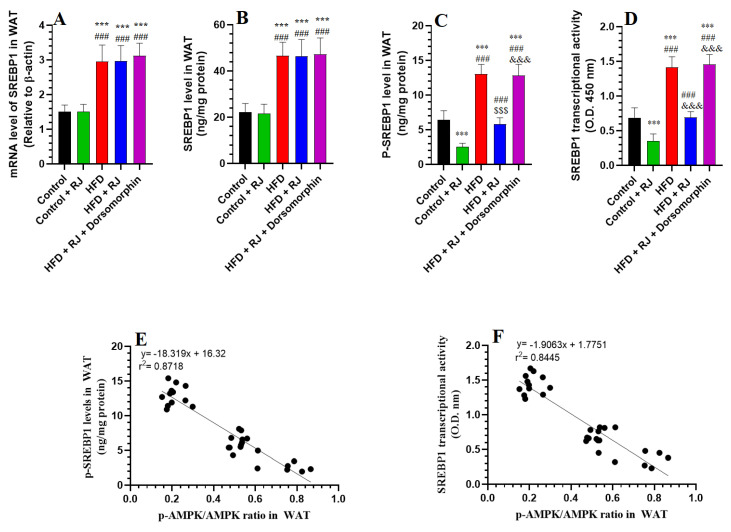
mRNA levels (**A**), total levels (**B**), phosphorylated levels (**C**), and transcriptional activity (**D**) of SREBP1 and their correlation with the phosphorylated levels of AMPK (**E**,**F**) in the white adipose tissue (WAT) of all groups of rats. Data are given as the means ± SD of 6 rats/group. ***: significantly different vs. control (*p* < 0.001); ###: significantly different vs. control + RJ (*p* < 0.001); $$$: significantly different vs. HFD (*p* < 0.001); and &&&: significantly different vs. HFD + RJ (*p* < 0.001). RJ: royal jelly; HFD: high-fat diet. Dorsomorphin: an AMPK inhibitor.

**Figure 6:**
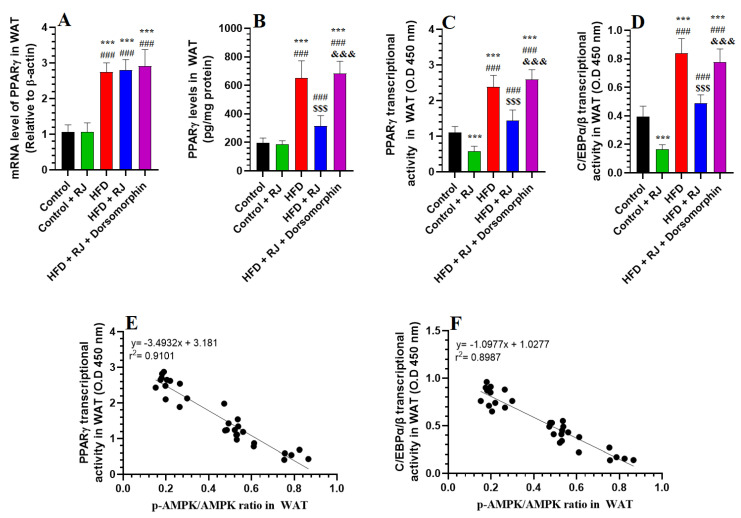
mRNA levels (**A**), total protein levels (**B**), nuclear transcriptional activity of PPARγ (**C**) and nuclear transcriptional activity of C/EBPαβ (**D**) and their respective correlation of the phosphorylation ratio of AMPK (**E**,**F**) in the white adipose tissue (WAT) of all groups of rats. Data are given as the means ± SD of 6 rats/group. ***: significantly different vs. control (*p* < 0.001); ^###^: significantly different vs. control + RJ (*p* < 0.001); ^$$$^: significantly different vs. HFD (*p* < 0.001); and ^&&&^: significantly different vs. HFD + RJ (*p* < 0.001). RJ: royal jelly; HFD: high-fat diet. Dorsomorphin: an AMPK inhibitor.

In the WAT from rats fed with an HFD, there was a pronounced increase in both the mRNA and protein levels of SREBP1 and PPARγ, alongside elevated p-SREBP1 levels and enhanced transcriptional activities of SREBP1, PPARγ, and C/EBPα/β, compared to the control rats (Figure 5A–D and Figure 6A–D). Treatment with RJ, either alone or in combination with an HFD, did not significantly alter the mRNA and protein levels of SREBP1 and PPARγ. However, both the RJ-treated and HFD + RJ-treated groups exhibited significant decreases in their p-AMPK levels and the nuclear transcriptional activities of SREBP1, PPARγ, and C/EBPα/β relative to the control and HFD-obese rats (Figure 5A–D and Figure 6A–D). No significant differences were observed in the mRNA levels of SREBP1 and PPARγ or in the protein levels of SREBP1 among the HFD, HFD + RJ, and HFD + RJ + dorsomorphin groups (Figure 5A,B and Figure 6A). Nonetheless, the protein levels of PPARγ and p-AMPK, as well as the transcriptional activities of SREBP1, PPARγ, and C/EBPα/β, were significantly elevated in the WAT of the HFD + RJ + dorsomorphin-treated rats compared to the HFD-fed rats (Figure 5C,D and Figure 6B–D). A correlation analysis indicated very strong negative associations between AMPK and p-SREBP1, as well as the transcriptional activities of SREBP1, PPARγ, and C/EBPα/β (r^2^ = 0.9718, 0.8445, 0.9101, and 0.8987, respectively).

### 3.6. RJ Stimulates Adipose Tissue Lipolysis by Activating HSL and TGL and Upregulating PGC-1α

**Figure 7:**
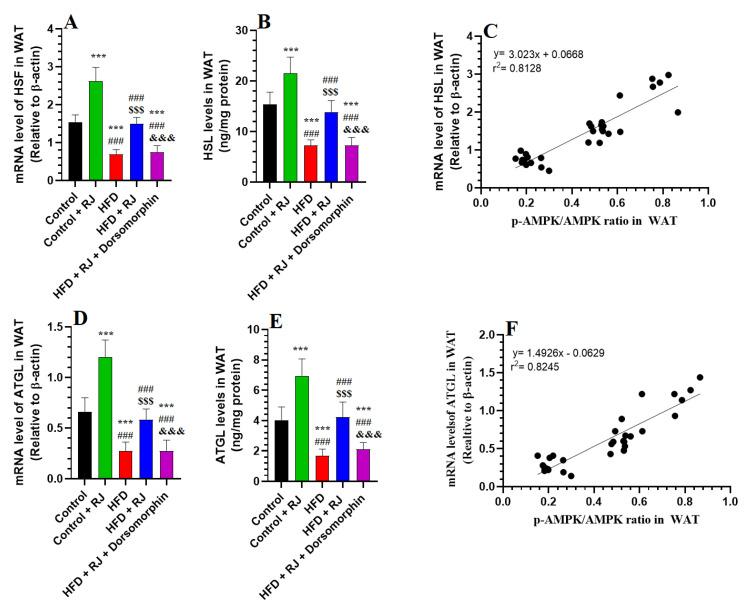
mRNA and total protein levels of hormone-sensitive lipase (HSL) (**A**,**B**) and adipose triglyceride lipase (ATGL) (**D**,**E**), as well as their corresponding and respective correlations with the phosphorylation levels of AMPK (**C**,**F**) in the white adipose tissue (WAT) of all groups of rats. Data are given as means ± SD of 6 rats/group. ***: significantly different vs. control (*p* < 0.001); ###: significantly different vs. control + RJ (*p* < 0.001); $$$: significantly different vs. HFD (*p* < 0.001); and &&&: significantly different vs. HFD + RJ (*p* < 0.001). RJ: royal jelly; HFD: high-fat diet. Dorsomorphin: an AMPK inhibitor.

**Figure 8:**
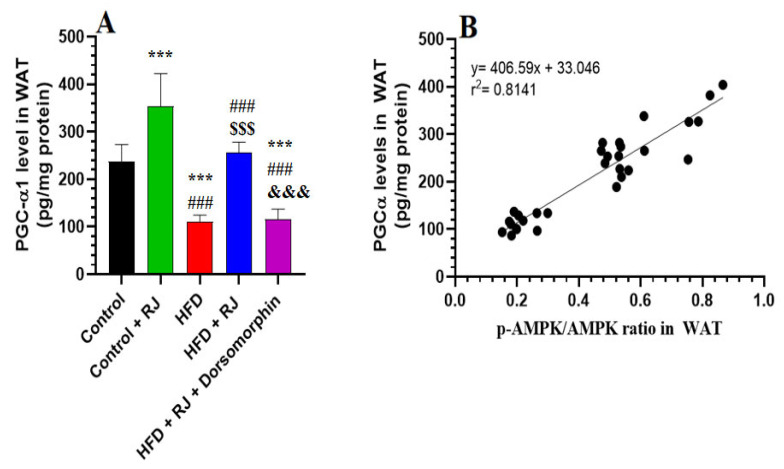
Levels of PGC-α1 (**A**) and its correlation with the ratio of the tissue phosphorylation ratio of AMPK (**B**) in the white adipose tissue (WAT) of all groups of rats. Data are given as the means ± SD of 6 rats/group. ***: significantly different vs. control (*p* < 0.001); ###: significantly different vs. control + RJ (*p* < 0.001); $$$: significantly different vs. HFD (*p* < 0.001); and &&&: significantly different vs. HFD + RJ (*p* < 0.001). RJ: royal jelly; HFD: high-fat diet. Dorsomorphin: an AMPK inhibitor.

In the WAT of rats subjected to an HFD, there was a marked elevation in both the mRNA and protein levels of HSL and ATGL, along with increased levels of PGC-α1, compared to the control rats (Figure 7A,B,D,E and Figure 8A,B). Similarly, the RJ and HFD + RJ treatment groups exhibited significantly higher mRNA and protein levels of HSL and ATGL in their WAT relative to the controls (Figure 7A,B,D,E). However, treatment with dorsomorphin in the HFD + RJ + dorsomorphin group led to a notable decrease in the WAT mRNA and protein levels of ATGL, HSL, and PGC-α1 compared to the HFD + RJ group (Figure 7A,B,D,E and Figure 8A,B). Despite this, there were no significant differences in the mRNA levels of HSL and ATGL, nor the protein levels of PGC-α1 between the HFD-obese rats and those treated with HFD + RJ + dorsomorphin. A strong positive correlation was observed between p-AMPK levels and the protein levels of HSL, ATGL, and PGC-α1, with correlation coefficients of r^2^ = 0.8128, 0.8245, and 0.8141, respectively (Figure 7C,F).

### 3.7. RJ Reduces the Size of the Adipocytes in the HFD-Fed Rats

**Figure 9:**
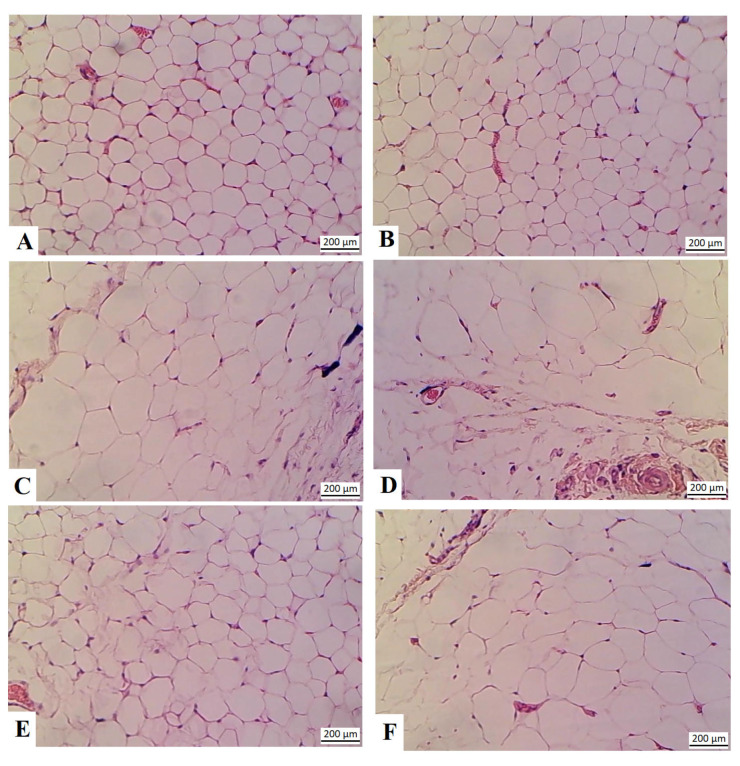
RJ reduces the size of the adipocytes in the HFD-fed rats. (**A**,**B**): control and control + RJ-treated rats; (**C**,**D**): HFD-fed rats; (**E**): HFD + RJ-treated rats; and (**F**): HFD + RJ+ dorsomporphin rats.

Adipocytes from the control and control + RJ-treated rats showed normal histological features of the adipose tissue, with rounded cells with sided nuclei (Figure 9A,B, respectively). The size of the adipocytes was obviously increased in the HFD-fed rats, with increased immune cell infiltration (Figure 9C,D). A significant reduction in the size of the adipocytes, with no evidence of the presence of immune cells in the section, was observed in the HFD + RJ-treated rats (Figure 9E). Similar increments in the size of the adipocytes with concomitant immune cell infiltration were observed in the adipose tissue of the HFD + RJ + dorsomporphin rats (Figure 9F).

## 4. Discussion

This study provides substantial evidence for the potent anti-obesity effects of RJ in a rodent model and elucidates the underlying molecular pathways involved. Our findings demonstrate that the daily administration of RJ at a dose previously shown to mitigate steatosis not only curtails the accumulation of body and adipose tissue mass, but also ameliorates insulin resistance, reduces lipogenesis, and enhances both lipolysis and fatty acid oxidation. The principal mechanisms appear to involve the downregulation of C/EBPα/β, PPARγ, and SREBP1, alongside the inhibition of adipose tissue inflammation and ACC. Furthermore, RJ induces the upregulation of PGC-1α, CPT-1, ATGL, and HSL. Importantly, these effects are contingent upon AMPK activation; the inhibition of AMPK abolishes any beneficial outcomes, resulting in biochemical, histological, and phenotypic profiles reminiscent of those observed in obese animal models.

Adipose tissue is pivotal in maintaining systemic homeostasis through its regulation of the energy, glucose, and lipid metabolisms [64]. This regulation occurs at both the local and systemic levels due to its dual role in fat storage and endocrine function [64]. The high-fat diet (HFD) model, frequently utilized to induce obesity in rodents, replicates the obesity phenotype observed in humans following prolonged exposure to a moderate energy intake [65,66]. This dietary regimen promotes adipose tissue expansion due to an increased caloric intake from fats, triggers inflammatory responses within the adipose tissue, and initiates IR [67]. Elevated levels of IL-6 and TNF-α correlate with an increased adipose tissue mass and IR in both humans and rodents [63,67]. Moreover, HFD-induced overnutrition and peripheral IR lead to elevated serum levels of TGs, CHOL, and LDL-c, while decreasing HDL-c levels, thus contributing to dyslipidemia and hepatic steatosis [68,69]. These findings were corroborated in our HFD-fed rat model, reinforcing the validity of our observations and the robustness of the model.

The administration of RJ over a 16-week period significantly attenuated final body weight gain, reduced the HOMA-IR index, and mitigated increases in glucose and insulin levels, while also improving dyslipidemia in the HFD-fed rats. In contrast, RJ exhibited no significant impact on the body weight, plasma glucose, or insulin levels of the control rats, although it markedly lowered their serum lipid levels, thereby affirming its hypolipidemic effects. These findings corroborate our previous evidence for RJ’s anti-obesity and anti-diabetic properties [51]. Moreover, RJ’s hypoglycemic, hypolipidemic, and insulin-sensitivity-enhancing effects have been documented in both diabetic humans (at doses of 1000–3000 mg/kg) and rodents (at doses of 100–300 mg/kg). RJ also significantly reduced the TNF and IL-6 levels in the adipose tissue of the HFD-fed rats, potentially attributable to its anti-inflammatory effects or its role in ameliorating insulin resistance. Inflammatory cytokines exacerbate adipose tissue insulin resistance, and conversely, IR can perpetuate inflammation [70]. RJ’s anti-inflammatory properties have been well-documented across various inflammatory models through the suppression of TNF-α, IL-6, and IL-1 synthesis [71]. Additionally, RJ’s anti-obesity and anti-diabetic effects are independent of changes in food intake or caloric consumption, as chronic RJ administration did not alter weekly food intake or lipid absorption [51]. Notably, RJ treatment significantly reduced anthropometric measures, including BMI, Lee index, adiposity index, and abdominal circumference, which are elevated in HFD-fed obese rodents, further validating its anti-obesity efficacy. RJ’s effectiveness in reducing body weight has also been observed in football players and individuals with established T2DM [45,46]. A recent meta-analysis of randomized controlled trials up to 2023 also corroborates RJ’s inhibitory effects on body weight, BMI, and fat mass [49].

To elucidate the molecular mechanisms underpinning RJ’s anti-obesity effects in HFD-fed rats, we assessed the expression and activity of the key genes and proteins involved in adipogenesis, lipolysis, and fatty acid oxidation. Under fed conditions, insulin and IGF-1 stimulate adipocyte differentiation by upregulating and activating several lipogenic transcription factors and genes [5]. Key adipogenic transcription factors include CREB, C/EBPα, C/EBPβ, PPARγ, and SREBP-1c [4,5]. PPARγ, a principal regulator of adipocyte differentiation, modulates metabolic processes such as insulin sensitivity, fatty acid uptake, lipid synthesis, and storage through the regulation of proteins including LPL, Ap2, CD36, and lipid-droplet-associated proteins [17,72]. CREB further stimulates differentiation by activating C/EBPβ, which enhances PPARγ expression and inflammation [5,73,74]. Conversely, SREBP-1c is a major transcription factor that inhibits β-oxidation and promotes lipogenesis by upregulating FA synthesis genes such as FAS and ACC [74]. The inhibitory effects of ACC on the carnitine cycle and β-oxidation are well-documented [75], and SREBP1 acts as an upstream activator of PPARγ [76]. In contrast, starvation conditions inhibit these pathways and activate lipolysis, with the TGs in the adipose tissue being hydrolyzed by ATGL to DAG, which is further broken down into fatty acids and glycerol by HSL [7].

Numerous novel targets have been identified that alleviate obesity by promoting lipolysis and fatty acid oxidation while inhibiting adipogenesis [10]. Our study observed marked increases in the mRNA, protein, and phosphorylation levels of SREBP1, alongside heightened phosphorylation of ACC and elevated mRNA, protein, and nuclear transcriptional activities of PPARγ and C/EBPα/β in the HFD-fed rats. These findings align with previous studies documenting similar changes in the expression and activation of these proteins [3,5]. RJ treatment significantly reduced the total protein levels and/or nuclear transcriptional activities of PPARγ, as well as the phosphorylated levels of ACC and SREBP1. Since RJ did not affect the mRNA or total protein levels of SREBP1 and PPARγ, this suggests that RJ’s mechanisms involve the degradation of PPARγ and reduced phosphorylation of SREBP1 and ACC, implicating an upstream kinase in this anti-adipogenic effect. Conversely, the RJ-treated HFD rats exhibited increased lipolysis, as evidenced by elevated ATGL and HSL levels and higher plasma glycerol and FFAs. The control rats administered with RJ also showed reductions in ATGL and HSF mRNA and protein levels and serum FFAs and glycerol, underscoring RJ’s potent lipolytic effects in both basal and obesity states.

Despite these findings, in vivo evidence for RJ’s effects on obesity and adipose tissue signaling pathways remains limited. Previous studies on rats have shown that RJ reduces body weight and enhances thermogenesis and WAT browning through the upregulation of CREBP1, UCP1, P38MAPK, BMP8B, and PRDM 16 [48]. Our study offers novel in vivo insights into RJ’s influence on key adipogenic and lipolytic transcription factors and enzymes. Additionally, RJ has been shown to mitigate hepatic lipogenesis by downregulating SREBP1 [51], and in vitro studies have demonstrated that 10-HAD, a compound isolated from RJ, reduces fat accumulation and inhibits SREBP1, FABP4, and C/EBPα in 3T3-L1 cells [77].

PGC-1α is a critical regulator of the lipid metabolism and thermogenesis across the liver, heart, muscle, and adipose tissue [78]. It suppresses TG synthesis and storage while stimulating FA oxidation by modulating PPARα and PPARγ, enhancing mitochondrial biogenesis, peroxisome activity, and the oxidation of long-chain FAs [78,79]. Furthermore, PGC-1α exerts antioxidant and anti-inflammatory effects, improving IR in the adipose tissue and muscle by inhibiting NF-κB and enhancing antioxidant pathways [79,80]. The reduced PGC-1α levels in HFD-fed rats correlate with obesity, BMI, inflammation, oxidative stress, and adipose tissue mass, while its activation confers protective benefits [81,82,83]. Our study revealed a significant increase in the PGC-1α mRNA levels in the adipose tissue of the HFD-fed obese rats, suggesting that RJ may enhance adipogenesis, lipolysis, and FA oxidation by upregulating PGC-1α and augmenting its signaling. This finding, along with RJ’s stimulatory effect on PGC-1α and UCP1, adds a novel dimension to our understanding of RJ’s metabolic and protective roles.

To delineate the precise molecular mechanisms underlying RJ’s anti-obesity effects, we focused on AMPK, given its central role in regulating adipogenesis, thermogenesis, lipolysis, and fatty acid oxidation [3]. AMPK inhibits FFA, triglyceride, and cholesterol synthesis by downregulating FAS, ACC1, and SREBP-1c, while promoting mitochondrial biogenesis and FA oxidation through PGC-1α activation [5,16,84]. In the adipose tissue, AMPK represses adipogenesis and pre-adipocyte differentiation by downregulating aP2, C/EBPα, C/EBPβ, and PPARγ and promotes autophagy and the apoptosis of adipocytes [3,5,85]. Additionally, AMPK enhances lipolysis by activating ATGL and HSL and modulates inflammation and thermogenesis [16,86,87,88]. Notably, AMPK activity was found to be negatively correlated with p-SREBP1 and the transcriptional activities of PPARγ, SREBP1, and C/EBPα, while positively correlating with PGC-1α, ATGL, and HSL levels. Importantly, the beneficial effects of RJ on adiposity markers, insulin resistance, and adipogenic and lipolytic markers were completely negated by AMPK inhibition, emphasizing AMPK’s pivotal role in mediating RJ’s anti-obesity, anti-adipogenic, and lipolytic effects. This is consistent with recent findings highlighting RJ’s potential to activate AMPK in various tissues and its ability to alleviate fasting hyperglycemia and improve IR through AMPK activation in the muscles and liver [50]. RJ’s prevention of NAFLD through the AMPK/SREBP1 axis further supports this [51].

The precise mechanism by which RJ activates AMPK in the adipose tissue remains elusive. Adiponectin and leptin, key adipose-derived factors, are known to influence AMPK activation. Leptin levels are elevated and leptin resistance is observed in obesity, while adiponectin levels are diminished. RJ treatment notably increased the adiponectin levels and reduced the elevated leptin levels in the HFD-fed rats. Our previous research showed a dose-dependent increase in serum adiponectin levels with RJ treatment [51], suggesting that RJ may activate AMPK in the adipose tissue and liver through enhanced adiponectin release. However, AMPK activation is also modulated by other upstream kinases and proteins, including MO25, LKB1, TAK1, SIRT1, CaMKK, and STRAD. Further investigation into these potential mechanisms is warranted.

RJ is known for its rich array of bioactive compounds that may contribute to its anti-obesity effects. Among these, proteins such as major royal jelly proteins (MRJPs) have been shown to improve insulin sensitivity and reduce obesity-related inflammation, highlighting their potential role in weight management [89]. Additionally, RJ contains unique lipids, including conjugated linoleic acid (CLA), which has been associated with reduced fat accumulation and improved body composition by inhibiting adipogenesis and promoting lipolysis [90]. The flavonoids present in RJ, such as quercetin and kaempferol, influence adipocyte differentiation and fat metabolism through their anti-inflammatory and antioxidant properties, thereby potentially reducing obesity risk [91]. Furthermore, the polyphenols in RJ, including various phenolic acids and flavonoids, modulate the lipid metabolism and enhance energy expenditure, contributing to obesity prevention [92,93]. These compounds act on multiple metabolic pathways, such as inhibiting lipogenic enzymes and promoting fatty acid oxidation. Integrating these insights into our discussion underscores the multifaceted roles of RJ’s bioactive compounds in combating obesity and suggests pathways for future research to explore their mechanisms further.

On the other hand, royal jelly (RJ) contains glucose, which can contribute to caloric intake if consumed in excessive amounts. However, research indicates that a moderate consumption of RJ is generally safe and beneficial, without leading to significant weight gain. RJ’s overall caloric contribution is relatively low compared to other dietary sources [94,95]. Studies suggest that a moderate intake of RJ does not adversely affect the glucose metabolism when included as part of a balanced diet [96]. The recommended daily dosage of RJ typically ranges from 200 mg to 300 mg, a range associated with beneficial effects on metabolic health and body weight without significant adverse effects [97,98]. For instance, Lee and Kim [99] found that daily supplementation with 300 mg of RJ for 12 weeks led to significant improvements in insulin sensitivity and reductions in body fat in overweight individuals. Thus, a moderate consumption of RJ, within these recommended amounts, is unlikely to contribute to obesity and can be part of a healthy diet. Updated evidence supports that RJ can be safely consumed for its potential anti-obesity benefits, provided that it is used in moderation and as part of a balanced dietary regimen [93].

## 5. Conclusions

The findings of this study are novel and conclusively show that RJ is an effective anti-obesity diet additive that can reduce the risk of obesity and its diabetic complications. Its protection mechanism involves AMPK signaling, which stimulates lipolysis and inhibits adipogenesis. Further examinations of the other upstream mechanisms that regulate AMPK, as well as more clinical trials in obese and diabetic individuals, are needed to confirm these effects.

## 6. Study Limitations

This study has several limitations that should be considered when interpreting the results. First, the use of only ELISAs for the protein analysis, due to constraints in funding and resources, limited our ability to conduct Western blotting, which could have provided more detailed information on protein expression and phosphorylation states. Western blot analysis is often considered to be the gold standard for such assessments, and its absence may have restricted the depth of our molecular characterization. Additionally, the study did not include measurements of perilipin, an important lipid droplet biomarker, due to time and financial constraints. The lack of this analysis means that our findings on lipid metabolism and adipogenesis may be incomplete. The study focused on a specific set of adiposity markers; therefore, a broader biomarker analysis could provide a more comprehensive understanding of the anti-obesity effects of royal jelly. Furthermore, the investigation was limited to a predetermined dosage and duration of royal jelly administration, which may not fully capture the long-term effects or optimal dosing needed for human consumption. The study’s design did not explore the effects of different royal jelly formulations or potential interactions with other dietary components, which could influence its efficacy and safety profile. Lastly, while the study utilized a well-established animal model, there may be limitations in translating these findings directly to human populations. Further research is needed to validate these results in clinical settings and to explore the underlying mechanisms more thoroughly. These limitations highlight areas for future research to build on our findings and address these gaps in knowledge.

## Figures and Tables

**Table 1:** Changes in anthropometrical markers among all groups of rats.

	Control	Control + RJ	HFD	HFD + RJ	HFD + RJ + Dorsomorphin
Final body weights	505 ± 25.4	494 ± 31.4	642 ± 47.8 *** ^###^	499 ± 38.5 ^$$$^	634 ± 42.3 *** ^### &&&^
Body length (cm)	27.73 ± 0.66	27.03 ± 0.8	27.3 ± 0.5	26.99 ± 0.55	27.0 ± 0.87 *** ^### &&&^
BMI	0.65 ± 0.04	0.62 ± 0.4	0.88 ± 0.1 *** ^###^	0.67 ± 0.03 ^$$$^	0.87 ± 0.08 *** ^### &&&^
AC (cm)	14.4 ± 0.89	15.1 ± 1.1	20.8 ± 1.8 *** ^###^	16.4 ± 1.3 ^$$$^	21.3 ± 1.6 *** ^### &&&^
Lee index	287.3 ± 12.2	293.4 ± 10.5	321.3 ± 13.7 *** ^###^	291.6 ± 11.4 ^$$$^	318.4 ± 12.8 *** ^### &&&^

Data are given as the means ± SD of 6 rats/group. ***: significantly different vs. control (*p* < 0.001); ###: significantly different vs. control + RJ (*p* < 0.001). $$$: significantly different vs. HFD (*p* < 0.001); and &&&: significantly different vs. HFD + RJ (*p* < 0.001). RJ: royal jelly; HFD: high-fat diet; BMI: body mass index; AC: abdominal circumference. Dorsomorphin: an AMPK inhibitor.

**Table 2:** Changes in the weights of the selected fat pads and the adiposity index (AI) among all groups of rats.

	Control	Control + RJ	HFD	HFD + RJ	HFD + RJ + Dorsomorphin
Mesenteric fat (g)	3.2 ± 0.35	2.9 ± 0.39	6.8 ± 0.52 *** ^###^	3.5 ± 0.46 ^# $$$^	6.4 ± 0.71 *** ^### &&&^
Retroperitoneal fat (g)	1.41 ± 0.12	1.22 ± 0.18	4.8 ± 0.39 *** ^###^	1.87 ± 0.14 * ^# $$$^	4.4 ± 0.61 *** ^### &&&^
Parametrial fat (g)	0.38 ± 0.05	0.31 ± 0.3	0.55 ± 0.03 * ^#^	0.58 ± 0.05 * ^#^	0.51 ± 0.05 * ^#^
Perirenal fat (g)	1.94 ± 0.21	2.11 ± 0.26	5.2 ± 0.48 *** ^###^	2.6 ± 0.51 ** ^# $$$^	4.9 ± 0.07 *** ^### &&&^
subcutaneous fat (g)	3.8 ± 0.05	3.3 ± 0.07	6.1 ± 0.08 *** ^###^	3.5 ± 0.07 ^$$$^	6.7 ± 0.08 *** ^### &&&^
Total fat weight (g)	10.65 ± 0.83	9.95 ± 0.78	22.93 ± 1.6 *** ^###^	12.82 ± 1.1 ^# $$$^	23.6 ± 1.96 *** ^### &&&^
AI	2.07 ± 0.14	1.93 ± 0.23	3.48 ± 0.26 *** ^###^	2.47 ± 0.34 * ^# $$$^	3.26 ± 0.23 *** ^### &&&^

Data are given as the means ± SD of 6 rats/group. *, **, and ***: significantly different vs. control (*p* < 0.05, 0.01, and 0.001); ^#^ and ^###^: significantly different vs. control + RJ (*p* < 0.05 and 0.001); ^$$$^: significantly different vs. HFD (*p* < 0.001); and ^&&&:^ significantly different vs. HFD+ RJ (*p* < 0.001). RJ: royal jelly; HFD: high-fat diet. Dorsomorphin: an AMPK inhibitor.

**Table 3:** Biochemical analysis in the plasma and serum of all groups of rats.

	Control	Control + RJ	HFD	HFD + RJ	HFD + RJ + Dorsomorphin
Fasting glucose (mg/dL)	101.6 ± 7.5	108.3 ± 12.6	185.4 ± 15.4 *** ^###^	131.3 ± 11.8 * ^# $$$^	186.3 ± 17.5 *** ^### &&&^
Fasting Insulin (ng/mL)	3.9 ± 0.26	4.2 ± 0.8	6.7 ± 0.7 *** ^###^	5.1 ± 0.36 * ^# $$$^	6.8 ± 0.6 *** ^### &&&^
HOMA-IR	0.95 ± 0.14	1.01 ± 0.26	3.09 ± 0.42 *** ^###^	1.62 ± 0.0.21 * ^# $$$^	3.18 ± 0.39 *** ^### &&&^
Serum-free fatty acids (μmol/L)	334.1 ± 39	268.4 ± 24 **	1098.4 ± 107 *** ^###^	383.1 ± 46 * ^### $$$^	1109.4 ± 106 *** ^### &&&^
Serum glycerol (μmol/L)	56.5 ± 4.8	88.8 ± 3.4 **	178.8 ± 14.7 *** ^###^	303.3 ± 5.8 * ^### $$$^	147.3 ± 15.9 *** ^### &&&^
Serum Leptin (ng/mL)	30.2 ± 4.7	27.8 ± 3.4	71.5 ± 6.2 *** ^###^	36.5 ± 4.8 * ^# $$^	76.5 ± 8.1 *** ^### &&&^
Serum adiponectin (μg/mL)	35.6 ± 3.2	47.6 ± 4.8 **	15.8 ± 1.7 *** ^###^	25.6 ± 2.9 * ^# $$^	13.9 ± 1.4 *** ^### &&&^
Serum Triglycerides (mg/dL)	46.4 ± 4.9	31.6 ± 3.8 ***	114.7 ± 19.9 *** ^###^	56.4 ± 4.9 * ^# $$^	105.5 ± 10.1 *** ^### &&&^
Serum Cholesterol (mg/kg)	98.5 ± 8.6	80.1 ± 7.1 ***	218.2 ± 18.9 *** ^###^	94.5 ± 9.7 ^## $$$^	227.3 ± 21.8 *** ^### &&&^
Serum LDL-c (mg/dL)	33.9 ± 2.9	26.2 ± 2.1 *	113.4 ± 11.7 *** ^###^	40.3 ± 3.9 * ^## $$$^	103.5 ± 9.8 *** ^### &&&^

Data are given as means ± SD of 6 rats/group. *, ** and ***: significantly different vs. control (*p* < 0.05 and 0.001); ^#^, ^##^, and ^###^: significantly different vs. control + RJ (*p* < 0.05, 0.01, and 0.001); ^$$^,^$$$^: significantly different vs. HFD (*p* < 0.001); and ^&&&^: significantly different vs. HFD+ RJ (*p* < 0.001). RJ: royal jelly; HFD: high-fat diet. Dorsomorphin: an AMPK inhibitor.

## Data Availability

The datasets used and analyzed in the current study are available from the corresponding author upon reasonable request.
